# PHGDH Promotes Synovial Aggression and Inflammation via Upregulating ADRA2A Expression in Rheumatoid Arthritis

**DOI:** 10.3390/cells15161501

**Published:** 2026-08-20

**Authors:** Kai Sun, Ting Liu, Xuanxian Xu, Huan Dong, Huijuan Hu, Chenxi Peng, Xiaofan Ge, Liuqin Liang, Youjun Xiao, Hanshi Xu, Qian Qiu

**Affiliations:** 1 Department of Rheumatology and Immunology, The First Affiliated Hospital, Sun Yat-Sen University, No.58 Zhongshan Er Road, Guangzhou 510080, China; sunk29@mail2.sysu.edu.cn (K.S.); liut98@mail2.sysu.edu.cn (T.L.); dongh29@mail2.sysu.edu.cn (H.D.); huhj29@mail2.sysu.edu.cn (H.H.); pengchx5@mail2.sysu.edu.cn (C.P.); gexf3@mail2.sysu.edu.cn (X.G.); lliuq@mail.sysu.edu.cn (L.L.); 2 Department of Anesthesiology, The First Affiliated Hospital, Sun Yat-Sen University, No. 58 Zhongshan Er Road, Guangzhou 510080, China; xuxx65@mail.sysu.edu.cn

**Keywords:** rheumatoid arthritis, fibroblast-like synoviocytes, PHGDH, aggression, inflammation

## Abstract

**Highlights:**

**What are the main findings?**

- PHGDH promotes RA FLS aggression and inflammation through ADRA2A.
- Local PHGDH knockdown alleviates arthritis in CIA rats.

**What are the implications of the main findings?**

- The PHGDH–ADRA2A axis contributes to pathogenic FLS activation.
- PHGDH represents a potential therapeutic target for RA.

**Abstract:**

Objectives: The role of Phosphoglycerate Dehydrogenase (PHGDH), the first key enzyme in the serine biosynthesis pathway, is important in controlling cancer survival; however, its role in rheumatoid arthritis (RA) remains unknown. Here, we investigated the functional involvement of PHGDH in RA pathogenesis, as well as its underlying molecular mechanisms. Methods: mRNA and protein expression in RA fibroblast-like synoviocytes (FLS) was measured by RT-qPCR and Western blot, respectively. Immunohistochemistry (IHC) was used to detect the protein expression in RA synovium. Cellular and tissue localization of the protein was assessed using IHC and immunofluorescence. The functional role of PHGDH in RA FLS was evaluated using multiple approaches: Transwell assays to assess cell migration and invasion, Annexin V/PI staining to detect apoptosis, and EdU assays to measure cell proliferation. Key downstream targets of PHGDH were identified via RNA sequencing (RNA-seq). The therapeutic potential of PHGDH targeting was further assessed in a rat collagen-induced arthritis (CIA) model following intra-articular administration of PHGDH-shRNA. Results: PHGDH expression was markedly elevated in RA synovial tissues and FLS. Functionally, knockdown of PHGDH suppressed proliferation, migration, invasion, and inflammatory cytokine production of RA FLS. Mechanistic studies revealed that PHGDH exerts its effects, at least in part, by inhibiting the expression of adrenoceptor alpha 2A (ADRA2A). The therapeutic relevance of these findings was further supported by in vivo experiments, where intra-articular delivery of PHGDH-shRNA significantly ameliorated the severity of arthritis in rats with CIA. Conclusions: Our results indicate that the PHGDH-ADRA2A axis critically drives the inflammatory and aggressive phenotype of RA FLS, suggesting that PHGDH might be as a promising therapeutic target for RA.

## 1. Introduction

Rheumatoid arthritis (RA) is a chronic, systemic autoimmune disease characterized by persistent joint inflammation, leading to irreversible joint damage. The pathological hallmarks of RA include synovitis, pannus formation, and the progressive destruction of cartilage and bone [1]. Despite considerable progress in the treatment of RA, a substantial proportion of patients do not achieve an adequate clinical response, underscoring the need for a deeper understanding of its pathogenesis to inform the development of novel targeted therapies.

Fibroblast-like synoviocytes (FLS) from RA patients display a persistently activated “tumor-like” phenotype, marked by robust secretion of inflammatory cytokines and matrix metalloproteinases (MMPs), as well as resistance to apoptosis, hyperproliferation, and increased invasiveness [2]. Based on their anatomical distribution, RA FLS can be classified into two subsets: the lining-layer PDPN^+^THY1^−^ population and the sublining-layer PDPN^+^THY1^+^ population, which display distinct functional phenotypes and exert differential effects on synovial structure and pathology [3]. Given the pivotal role of FLS in joint destruction in RA, and the fact that current systemic anti-inflammatory treatments fail to completely control this destruction [2], targeting aberrant FLS behavior has been proposed as a promising therapeutic approach [4,5]. Nevertheless, the mechanisms that drive the distinct inflammatory and invasive properties of RA-derived FLS compared with normal FLS remain largely unknown.

Phosphoglycerate dehydrogenase (PHGDH) serves as the rate-limiting enzyme that initiates the de novo serine biosynthesis pathway, catalyzing the conversion of 3-phosphoglycerate (3-PG) to 3-phosphohydroxypyruvate [6]. Through this pathway, PHGDH drives tumor initiation and progression in multiple cancers, including hepatocellular carcinoma [6], gastric cancer [7], and breast cancer [8]. Intriguingly, recent evidence has revealed an additional transcriptional regulatory function for PHGDH, implicating it in amyloid pathology in Alzheimer’s disease [7]. These observations suggest that PHGDH exerts transcriptional regulatory functions independent of its canonical metabolic activity. Nevertheless, its involvement in the pathogenesis of RA remains unexplored.

In this study, we found that PHGDH was upregulated in FLS and synovial tissue from patients with RA. We further investigated its contribution to the aberrant biological behavior of RA FLS, including migration, invasion, inflammation, and proliferation. Mechanistically, ADRA2A was identified as a potential downstream mediator of PHGDH in regulating these cellular events. Overall, this study aimed to elucidate the role of PHGDH in RA and to define the regulatory link between PHGDH and ADRA2A, positioning PHGDH as a promising therapeutic target for the disease.

## 2. Materials and Methods

### 2.1. Synovial Tissues and FLSs Culture

The diagnosis of RA was based on the 2010 American College of Rheumatology/EULAR classification criteria [9]. Synovial tissues were obtained from 20 patients with active RA (17 women and 3 men; median age: 58 (range: 41–77) years) who underwent knee synovectomy. The detailed demographics and clinical characteristics of these patients with RA are shown in Table S1. Osteoarthritis (OA) synovial tissues were harvested from sex- and age-matched patients who underwent arthroplasty for end-stage primary OA (7 women and 3 men; median age: 58 (52–81) years).

The obtained synovial tissue was washed three times with sterile phosphate-buffered saline (PBS, pH 7.4), after which adherent fat, fascia, and muscle tissue were removed. The tissue was cut into small pieces, evenly spread in a 60 mm cell culture dish, and placed in a 37 °C, 5% CO_2_ incubator for 2 h to enhance adhesion. DMEM complete medium containing 10% fetal bovine serum (FBS) and 1% penicillin-streptomycin was then added, and the culture was maintained under the same conditions until typical spindle-shaped cells emerged. Cells at passages 4–6 were used for the following cell experiments. The human study protocol was approved by the Ethics Committee of the First Affiliated Hospital of Sun Yat-sen University and was in accordance with the Declaration of Helsinki, (No.[2021]042, Date: 16 June 2023). All patients involved in the study provided written informed consent.

### 2.2. Quantitative Real-Time PCR

Cells were lysed with TRIzol, and the lysate was collected. RNA was extracted via chloroform extraction, precipitated with isopropanol, washed with 75% ethanol, and completely dissolved in an appropriate volume of DEPC-treated water. RNA concentration and purity were assessed using a NanoDrop microvolume spectrophotometer (Thermo Fisher Scientific, Wltham, MA, USA). The cDNA synthesis reaction mixture was prepared following the instructions of the Evo M-MLV reverse transcription kit, gently mixed, briefly centrifuged, and then subjected to reverse transcription in a thermal cycler under the following conditions: 37 °C for 15 min, followed by 85 °C for 5 s. The qPCR reaction mixture was prepared according to the SYBR Green qPCR kit instructions, and two-step qPCR amplification was performed. After amplification, the data were exported, and the relative expression levels of target genes were calculated using the 2^−ΔΔCT^ method (ΔΔCt = ΔCt sample − ΔCt calibrator). The primers are listed in Table S2.

### 2.3. Western Blot

FLS were incubated with RIPA buffer on ice for 5 min and collected using a cell scraper. After centrifugation at 12,000 rpm for 15 min at 4 °C, the protein-containing supernatants were retained. Protein concentrations were measured using a bicinchoninic acid assay, and samples were adjusted to equal concentrations before being mixed with loading buffer and heat-denatured. Equal amounts of protein were separated by sodium dodecyl sulfate-polyacrylamide gel electrophoresis (SDS-PAGE) and transferred onto Immun-Blot PVDF membranes (Bio-Rad, Hercules, CA, USA). The membranes were blocked with 5% non-fat milk and then incubated with primary antibodies overnight at 4 °C. Following incubation, the membranes were washed three times with TBST on a shaker for 15 min per wash, and then probed with secondary antibodies for 1 h at room temperature. Protein bands were visualized using ECL (Millipore, Burlington, MA, USA). The primary antibodies used were anti-PHGDH (14719-1-AP, Proteintech, Wuhan, China), anti-ADRA2A (14266-1-AP, Proteintech, Wuhan, China), and anti-β-actin (66009-1-Ig, Proteintech, Wuhan, China). Images were developed and captured using a chemiluminescence imaging system, and grayscale values were statistically analyzed using ImageJ (verion 1.54p) software.

### 2.4. RNA Interference

The specific small interfering RNA (siRNA) targeting PHGDH and ADRA2A was purchased from Beijing Tsingke Biotechnology Co., Ltd (Beijing, China). Transient transfection was performed using CALNP^TM^ RNAi in vitro (Beijing D-Nano Pharmaceutical Technology Co., Ltd., Beijing, China) at a final siRNA concentration of 100 nmol/L when cells reached approximately 70% confluence. For each well of a 6-well plate, 1800 μL of DMEM high-glucose medium was mixed with 200 μL of the prepared siRNA-transfection complexes. The siRNA-transfection complexes were prepared according to the manufacturer’s instructions. Briefly, 1 μL siRNA solution was mixed with 7 μL Reagent A and 2 μL Reagent B, incubated at room temperature for 5 min, and then supplemented with 190 μL of DMEM to obtain the siRNA-transfection complex solution. After 24 h, the transfection mixture was replaced with DMEM high-glucose medium containing 10% fetal bovine serum, and cells were cultured for an additional 24–48 h prior to subsequent experiments. The target sequences of siRNAs are listed in Table S3.

### 2.5. Lentivirus and Adenovirus Infection

Lentiviral particles for ADRA2A overexpression (OE-ADRA2A) and PHGDH knockdown (shPHGDH) were obtained from Genechem (Shanghai, China). FLS were transduced with lentiviral particles at a multiplicity of infection (MOI) of 40 using HitransG P (Genechem, China), according to the manufacturer’s instructions. For in vivo experiments, adenoviral particles were administered via intra-articular injection in CIA rats. The target sequences for OE-ADRA2A and shPHGDH are listed in Table S3 and Sequence S1, respectively.

### 2.6. EdU Proliferation Assay

At approximately 80% confluence, 100 μL of DMEM medium containing 10 μmol/L EdU and 10% FBS was added to each well and incubated for 24 h. After washing three times with PBS, cells were fixed with 4% paraformaldehyde for 15 min at room temperature. The cells were then washed three times (5 min each) with wash buffer (PBS containing 3% BSA), permeabilized with permeabilization buffer (PBS containing 0.3% Triton X-100) for 15 min, and washed twice. The Click reaction mixture was prepared according to the manufacturer’s instructions (Click Reaction Buffer 430 μL, CuSO_4_ 20 μL, Azide 555 1 μL, Click Additive Solution 50 μL) and added to the wells for 30 min at room temperature in the dark. After removal of the reaction mixture, cells were washed three times, and nuclei were counterstained with DAPI for 10 min in the dark. Following three additional washes, EdU-positive cells were visualized under a fluorescence microscope and quantified.

### 2.7. Apoptosis Analysis

FLS grown in six-well plates were harvested at approximately 80% confluence using 0.25% EDTA-free trypsin. The cells were collected by centrifugation at 1500 rpm for 5 min and washed twice with chilled PBS. They were then resuspended in 1 × binding buffer at 5 × 10^6^ cells/mL.

A 100-μL aliquot of each cell suspension was incubated with 5 μL of Annexin V-AF647 and 5 μL of propidium iodide (PI) for 15 min at room temperature in the dark. Samples were analyzed by flow cytometry within 1 h of staining.

### 2.8. Transwell Migration and Invasion Assay

Cells were collected and resuspended at a concentration of 1 × 10^5^ cells/mL. The cell suspension (200 μL per well) was added to the upper chamber of Transwell inserts for the migration assay. The Transwell chamber was then incubated at 37 °C with 5% CO_2_ for 6–8 h. After incubation, the liquid in the upper chamber was removed, and the inserts were gently washed three times with PBS. Cells were fixed with 4% paraformaldehyde for 15 min at room temperature, followed by staining with crystal violet for 15 min. Non-migrated cells on the upper side of the membrane were carefully removed with a cotton swab. Images were captured from randomly selected fields under an optical microscope, and migrated cells were counted.

For the invasion assay, Transwell inserts were pre-coated with Matrigel. Briefly, Matrigel was diluted 1:20 in DMEM medium, and 50 μL of the diluted Matrigel was added evenly to the upper chamber, avoiding air bubbles. The coated inserts were then incubated in a cell culture incubator for at least 4 h to allow polymerization prior to cell seeding.

### 2.9. Transcriptome Sequencing and Bioinformatics Analysis

Total RNA was extracted using the TRIzol method. RNA concentration was measured with the Qubit RNA HS Assay Kit (Thermo Fisher Scientific, Eugene, OR, USA), and RNA quality was assessed by agarose gel electrophoresis. After quality control approval, libraries were constructed using the EpiTM mRNA Rapid Library Prep Kit and further validated with Qsep. High-throughput sequencing was performed on the SURFSeq 5000 platform (GeneMind, Shenzhen, China). Raw data were subjected to adapter trimming and quality filtering to generate clean reads. The clean reads were then aligned to the human reference genome GRCh38 (Ensembl) using Hisat2 (v2.1.0). Gene expression quantification was performed with featureCounts (v1.6.3). Differential expression analysis was conducted using the DESeq2 package in R (version 4.3.3), and pathway enrichment analysis was performed with the ReactomePA package. Results were visualized using the ggplot2 package to generate volcano plots and heatmaps.

### 2.10. Immunohistochemistry (IHC) and Immunofluorescence (IF) Staining

The paraffin sections were baked at 60–65 °C for 1–2 h, dewaxed in xylene twice for 10–15 min each, and rehydrated through a graded ethanol series (100%, 95%, 90%, 80%, 70%) for 3–5 min each, followed by a 5-min rinse in distilled water or PBS. Antigen retrieval was performed by heating the sections in retrieval buffer (citrate buffer, pH 6.0, or Tris-EDTA buffer, pH 9.0) to 95–100 °C for 10–20 min, followed by natural cooling to room temperature. Endogenous peroxidase activity was blocked using a peroxidase blocking reagent, and the sections were blocked with PBS containing 5% BSA for 30 min at room temperature. After removing the blocking solution, the sections were incubated with primary antibody working solution overnight at 4 °C in a humidified chamber, followed by incubation with secondary antibody working solution for 1 h at room temperature. Color development was achieved using 1× DAB working solution and monitored under a microscope. Sections were counterstained with hematoxylin for 30 s to 1 min, differentiated in hydrochloric acid-ethanol for approximately 10 s, and blued. Dehydration was carried out through a graded alcohol series (75%, 90%, and 95% ethanol for 5 min each, and absolute ethanol twice for 5 min each), followed by clearing in xylene (twice for 5 min each). After air-drying, sections were mounted with neutral resin, coverslipped, and observed under a microscope for image acquisition.

For immunofluorescence, following incubation with primary antibodies, fluorescence labeling and signal amplification were performed using the Tyramide Signal Amplification (TSA) plus Fluorescein System (Servicebio, Wuhan, China). Nuclei were counterstained with DAPI. Images were captured using a fluorescence microscope (Olympus BX53, Tokyo, Japan).

### 2.11. Single-Cell RNA Sequence (scRNA-seq) Analysis

Single-cell RNA sequencing data from rheumatoid arthritis synovial tissue were downloaded from the GEO database (GSE200815) and were analyzed using the Seurat R package. Cells with low gene numbers, extremely high gene numbers, or high mitochondrial gene percentages were removed for quality control. After normalization, highly variable genes were identified, followed by data scaling and principal component analysis. Cell clustering was performed using the FindNeighbors and FindClusters functions, and UMAP was used for dimensionality reduction and visualization.

Marker genes for each cluster were identified using FindAllMarkers. Cell types were manually annotated according to canonical marker genes, including fibroblasts, macrophages, T cells, B cells, plasma cells, proliferating T cells, and erythroid-like/contaminating cells. The expression distribution of PHGDH and ADRA2A was visualized using a UMAP feature plot or a jitter dot plot across different cell types.

### 2.12. Local Joint Knockdown of PHGDH in Rats with Collagen-Induced Arthritis (CIA)

Male Wistar rats were used to establish the CIA model as previously described [10]. Arthritis was induced by injecting 200 μL of an emulsified 1:1 mixture of bovine type II collagen (2 mg/mL; Chondrex, Woodinville, WA, USA) and complete Freund’s adjuvant (5 mg/mL; Chondrex, USA) at approximately 1.5 cm distal to the base of the tail. A booster injection (100 μL per rat, same concentration) was administered 7 days later. To achieve local knockdown of PHGDH in the joints, 50 μL of recombinant adenovirus (control vector or shPHGDH, 2 × 10^8^ PFU; Genechem, Shanghai, China) was administered via intra-articular injection on days 8 and 15 after primary immunization. Arthritis severity, including arthritis score, ankle circumference, and paw volume, was evaluated every other day after the booster immunization according to a previously described scoring system [11]. After the final assessment, all animals were euthanized by intraperitoneal injection of sodium pentobarbital. The animal study was approved by Animal Experimental Ethics Committee in Sun Yat-sen University (No.: 2026001888, Date: 5 June 2026).

### 2.13. Micro-Computed Tomography (CT)

Micro-CT imaging of the hind limbs was performed using a SkyScan 1276 system (Bruker, Bremen, Germany), followed by 3D reconstruction with 3-matic software (version 20.0).

### 2.14. Histological Changes in the CIA Model

Paraffin-embedded sections were dewaxed in xylene and rehydrated through a graded ethanol series, then subjected to hematoxylin-eosin (H&E) staining and Safranin O-Fast Green staining, respectively. HE staining was used to assess general tissue morphology and inflammatory infiltration, whereas Safranin O-Fast Green staining served to evaluate matrix changes in cartilage and bone tissues. Following staining, sections were dehydrated, cleared in xylene, mounted with neutral resin, and imaged under a light microscope.

### 2.15. Enzyme-Linked Immunosorbent Assay

FLS culture supernatants were collected and centrifuged at 1000× *g* for 10 min at 4 °C to remove cells and debris. The concentrations of human IL-1β, IL-6, IL-8, and CCL2 were measured using commercially available ELISA kits (Jianglai Biotechnology, Shanghai, China) according to the manufacturer’s instructions. Samples and standards were assayed in duplicate. Absorbance was measured at 450 nm using a microplate reader, and cytokine concentrations were calculated from standard curves using four-parameter logistic regression and corrected for the corresponding dilution factors.

### 2.16. Statistical Analysis

Statistical analyses were performed using GraphPad Prism (version 9.5.0). Quantitative data are reported as the mean ± standard deviation (SD), and in vitro experiments were independently repeated at least three times. Two-group comparisons were conducted using a two-tailed unpaired Student’s (*t*)-test for normally distributed data or a Mann–Whitney (U)-test for non-normally distributed data. Differences among more than two groups were assessed by one-way analysis of variance followed by Bonferroni’s multiple-comparison test. Statistical significance was defined as *p* < 0.05.

## 3. Results

### 3.1. PHGDH Expression Is Increased in FLS and Synovial Tissue from Patients with RA

**Figure 1:**
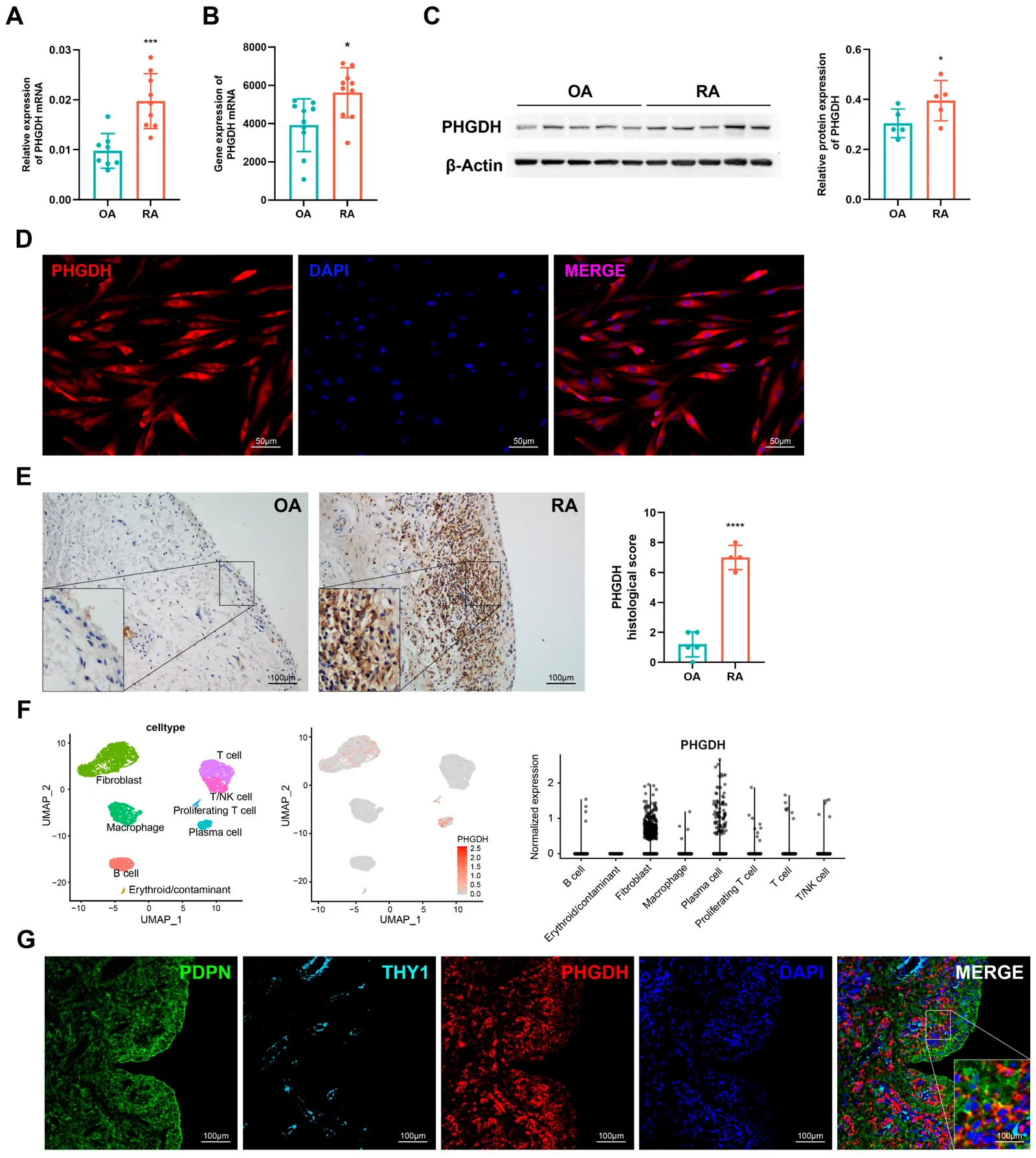
Elevated expression of PHGDH in fibroblast-like synoviocytes (FLS) and synovium from patients with rheumatoid arthritis (RA). (**A**) RT-qPCR showed an elevated mRNA level of PHGDH in FLS from 9 RA patients compared to 8 OA patients. (**B**) The elevated mRNA level of PHGDH in RA FLS (*n* = 10) was verified in a GEO database (GSE112656) compared with OA FLS (*n* = 10). (**C**) Western blot showed an elevated protein level of PHGDH in FLS from 5 RA patients compared to 5 OA patients. (**D**) Immunofluorescence cytochemistry (ICC) showed a predominant cytoplasm localization of PHGDH in RA FLS. (**E**) Immunohistochemistry (IHC) showed elevated expression of PHGDH in RA synovium (original magnification, ×200). (**F**) The UMAP feature plot and jitter dot plot showed that PHGDH was mainly expressed in plasma cells and FLS, as revealed by single-cell RNA sequencing analysis. (**G**) Multiplex immunofluorescence (IF) showed that PHGDH was localized in both the synovial lining and sublining layers of RA synovium (Original magnification, ×200). Data are presented as the means ± SD of at least 3 independent experiments. * *p* < 0.05, *** *p* < 0.001, and **** *p* < 0.0001 versus OA by Student’s *t*-test. PHGDH, Phosphoglycerate Dehydrogenase; FLS, fibroblast-like synoviocytes; RA, rheumatoid arthritis; OA, osteoarthritis; RT-qPCR, Quantitative real-time PCR; ICC, Immunofluorescence cytochemistry; IHC, immunohistochemistry; IF, immunofluorescence.

To characterize the expression pattern of PHGDH in RA, we first examined its expression levels in RA FLS. The results showed that PHGDH mRNA expression was significantly upregulated in RA FLS compared with osteoarthritis (OA) FLS (Figure 1A). Further analysis of the GSE112656 dataset revealed consistent differences in PHGDH expression between OA and RA, confirming the high mRNA expression of PHGDH in RA FLS (Figure 1B). Subsequently, we confirmed the high expression of PHGDH in RA FLS at the protein level by Western blotting (Figure 1C). Cell immunofluorescence staining showed that PHGDH was predominantly localized in the cytoplasm of RA FLSs (Figure 1D). To assess PHGDH expression in RA synovial tissue, immunohistochemistry was performed. PHGDH expression was found to be upregulated in RA synovial tissue compared with OA synovial tissue (Figure 1E). ScRNA-seq data indicated that PHGDH expression was mainly enriched in B cells and FLS (Figure 1F). Moreover, multiplex immunofluorescence staining was performed to characterize the spatial distribution of PHGDH within distinct fibroblast compartments of RA synovial tissue. Using PDPN and THY1 as markers to identify and characterize synovial fibroblast populations, PHGDH expression was detected in both the lining and sublining compartments (Figure 1G), indicating that PHGDH expression was not restricted to a single anatomical fibroblast niche.

### 3.2. PHGDH Regulates Multiple Pathogenic Behaviors of RA FLSs

**Figure 2:**
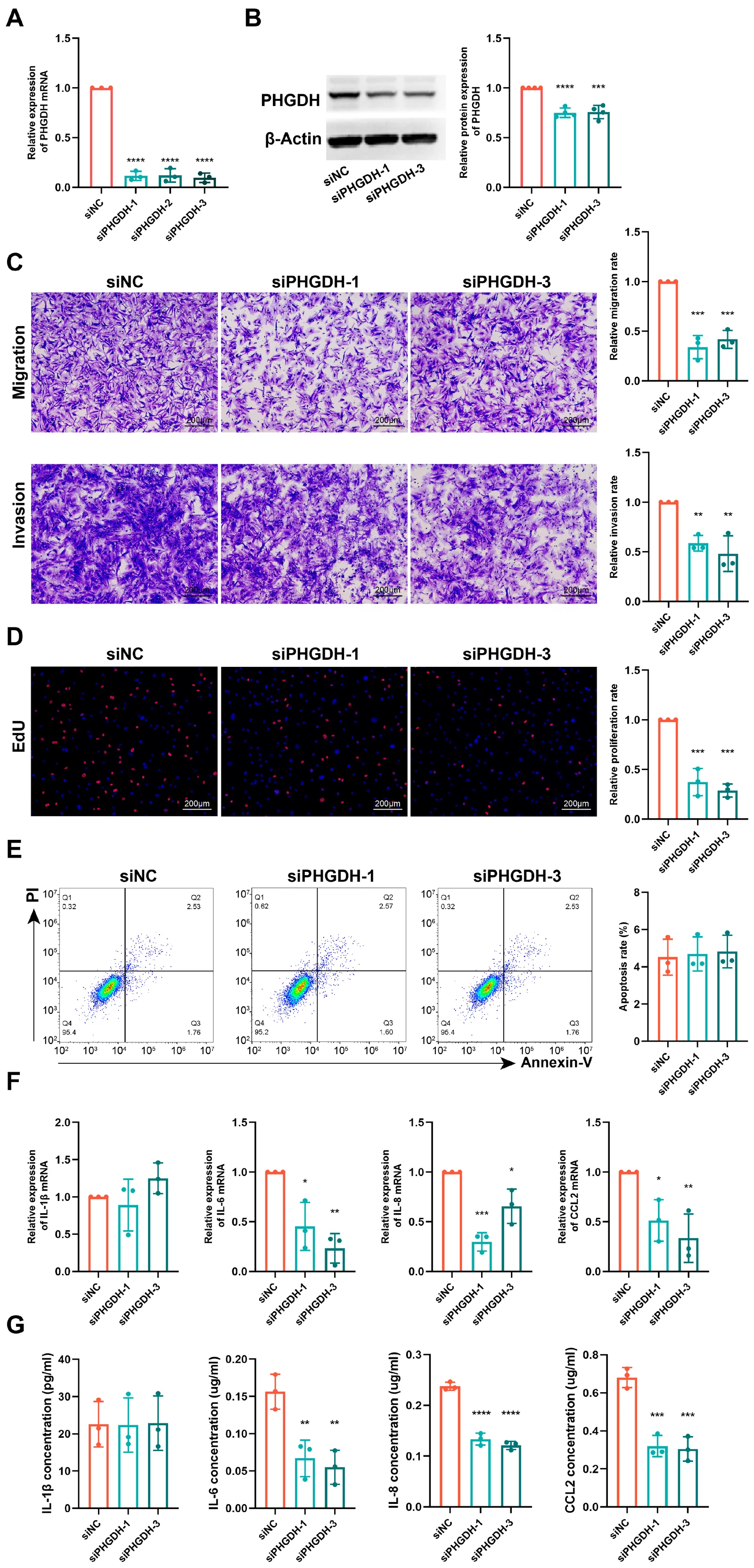
Effect of PHGDH knockdown on the biological functions of RA FLS. (**A**,**B**) The PHGDH-siRNA knockdown efficiency verification by RT-qPCR and Western blot. Two siRNAs (siPHGDH-1 and siPHGDH-3) with stable knockdown efficiency were selected for subsequent functional experiments. (**C**) Transwell assays were used to evaluate the chemotactic migration and invasion of RA FLS. Relative migration and invasion rates were determined by counting migrated or invaded cells in each group and normalizing them to the siNC group. Representative images and corresponding quantitative analyses are shown. Original magnification, ×100. (**D**) Cell proliferation was evaluated by EdU incorporation assays. Representative images of EdU-positive proliferating cells and DAPI-stained nuclei are shown, with corresponding quantification of proliferation rates in the right panel. Original magnification, ×100. (**E**) Cell apoptosis was assessed by PI/Annexin V staining followed by flow cytometry. Representative plots and corresponding quantification of apoptotic cells are shown. Apoptotic cells were defined as the sum of cells in the upper-right and lower-right quadrants. (**F**,**G**) RT-qPCR and ELISA were used to assess the mRNA and secretion levels of proinflammatory cytokines. Quantitative analyses of relative cytokine expression are shown. Data are presented as the means ± SD of at least 3 independent experiments. * *p* < 0.05, ** *p* < 0.01, *** *p* < 0.001, and **** *p* < 0.0001 versus siNC by Student’s *t*-test. PHGDH, Phosphoglycerate Dehydrogenase; RT-qPCR, Quantitative real-time PCR; NC, negative control; EDU, 5-ethynyl-2′-deoxyuridine; DAPI, 4′,6-Diamidino-2-phenylindole.

To investigate the effect of PHGDH on the functions of RA FLS, we designed three siRNAs and selected the two with the highest knockdown efficiency verified by RT-qPCR (Figure 2A) and Western blot (Figure 2B) for subsequent experiments. The migration and invasion of RA FLS, the key pathological processes driving cartilage destruction and bone erosion in RA, were measured using Transwell assay. Our results showed that PHGDH knockdown significantly inhibited the migration and invasion capabilities of RA FLS (Figure 2C). Given the critical role of the aberrantly enhanced proliferation and anti-apoptotic capacity of RA FLS in synovial hyperplasia, we performed EdU incorporation assays and flow cytometry and found that PHGDH knockdown markedly suppressed cell proliferation (Figure 2D) but had no significant effect on apoptosis (Figure 2E). Furthermore, considering that inflammatory cytokines are key mediators of synovial inflammation and joint destruction in RA, we examined the effect of PHGDH on the expression of major inflammation factors enriched in RA synovial microenvironment using RT-qPCR. The results showed that PHGDH knockdown downregulated the expression and secretion of IL-6, IL-8, and CCL2, but had negligible effects on the expression level of IL-1β (Figure 2F,G). In summary, PHGDH regulates the migration, invasion, proliferation, and inflammatory response of RA FLS, positioning it as a promising key target in the pathogenesis of RA.

### 3.3. ADRA2A Is a Potential Downstream Target of PHGDH

**Figure 3:**
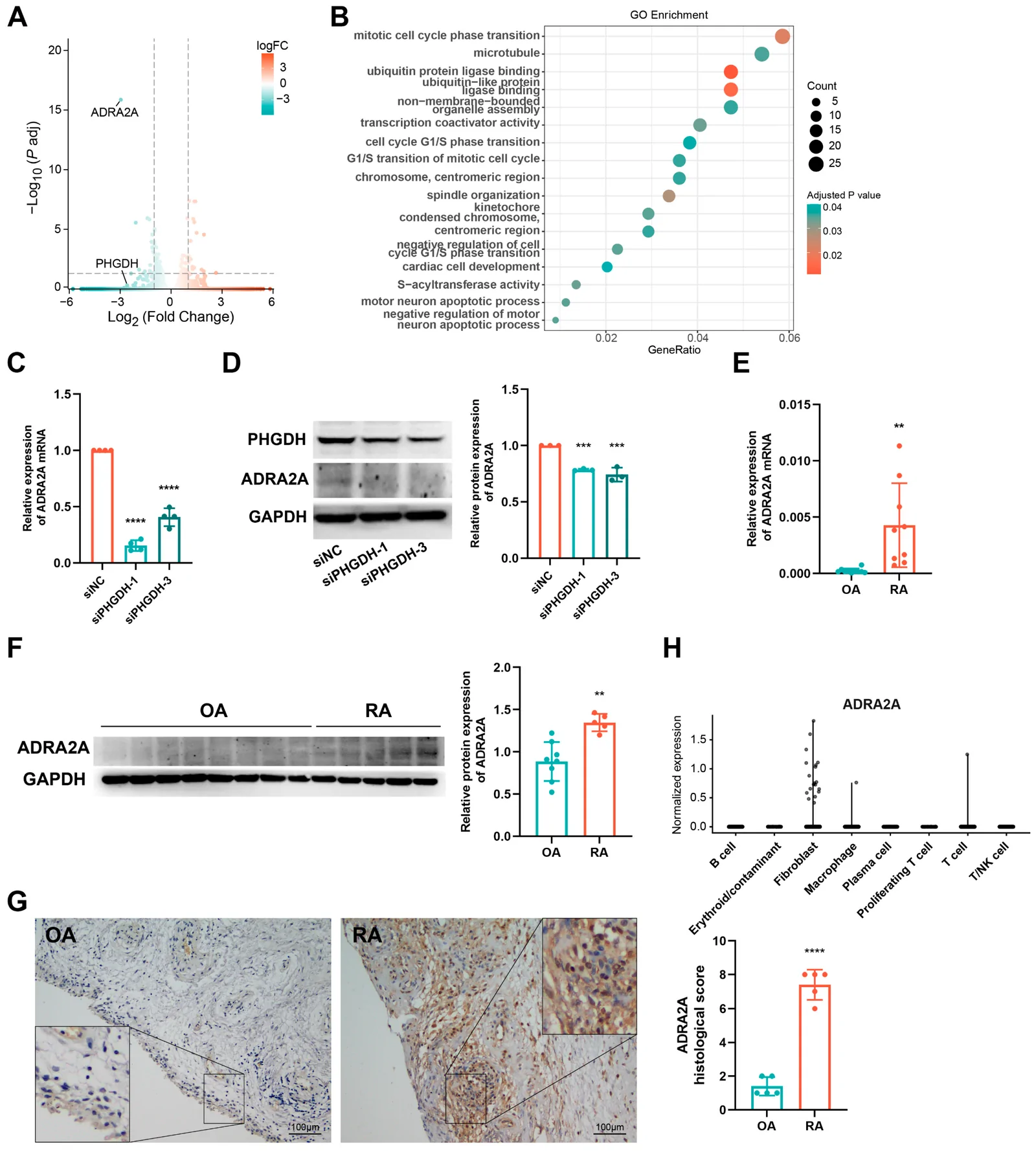
Identification of ADRA2A as a potential downstream target of PHGDH in RA FLS. (**A**) The volcano plot shows the distribution of differentially expressed genes (DEGs) between the siPHGDH and siNC groups (siPHGDH vs. siNC), with ADRA2A identified as the most markedly downregulated gene following PHGDH knockdown. (**B**) Dot plot of GO items show that mitotic cell cycle transition was the most significantly downregulated pathway following PHGDH knockdown. (**C**,**D**) Downregulated expression of ADRA2A after knockdown of PHGDH in RA FLS confirmed by RT-qPCR and Western blot. (**E**,**F**) Elevated ADRA2A expression was verified in RA FLS compared to OA FLS by RT-qPCR and Western blot. (**G**) IHC showed elevated ADRA2A expression in RA synovial tissues compared to OA synovial tissues. Original magnification, ×200. (**H**) The jitter dot plot showed that ADRA2A was mainly expressed in FLS, as revealed by single-cell RNA sequencing analysis. Data are presented as the means ± SD of at least 3 independent experiments. ** *p* < 0.01, *** *p* < 0.001, and **** *p* < 0.0001, versus siNC or OA by Student’s *t*-test. ADRA2A, Adrenoceptor Alpha 2A; PHGDH, Phosphoglycerate Dehydrogenase; DEGs, differentially expressed genes; RA, rheumatoid arthritis; FLS, fibroblast-like synoviocytes; OA, osteoarthritis; RT-qPCR, Quantitative real-time PCR; IHC, immunohistochemistry.

To explore the molecular mechanism by which PHGDH regulates the function of RA FLS, we performed RNA-seq analysis. The results showed that, compared with the siNC group, 541 genes were significantly upregulated and 537 genes were significantly downregulated in the siPHGDH group (Figure 3A). Further GO enrichment analyses revealed that the downregulated differentially expressed genes were primarily enriched in the mitotic cell cycle transition pathway, exhibiting suppressed G1/S cell cycle transition and indicating inhibition of cell proliferation (Figure 3B). These GO analysis results are largely consistent with the functional experimental data. Among the differentially expressed genes, *ADRA2A* showed the most significant differential expression (Figure 3A). To determine whether PHGDH exerts its regulatory effects through ADRA2A, we examined the effect of PHGDH knockdown on ADRA2A expression using RT-qPCR (Figure 3C) and Western blotting (Figure 3D). The results showed that PHGDH knockdown significantly downregulated ADRA2A expression, suggesting that ADRA2A might be a potential downstream target of PHGDH. Furthermore, to test whether this regulation is mediated by serine, we performed metabolite supplementation experiments. Our data showed that exogenous serine supplementation had a negligible effect on ADRA2A expression (Figure S1), indicating that PHGDH regulates ADRA2A through a serine-independent mechanism.

### 3.4. Role of ADRA2A in Regulating Functions of RA FLS

**Figure 4:**
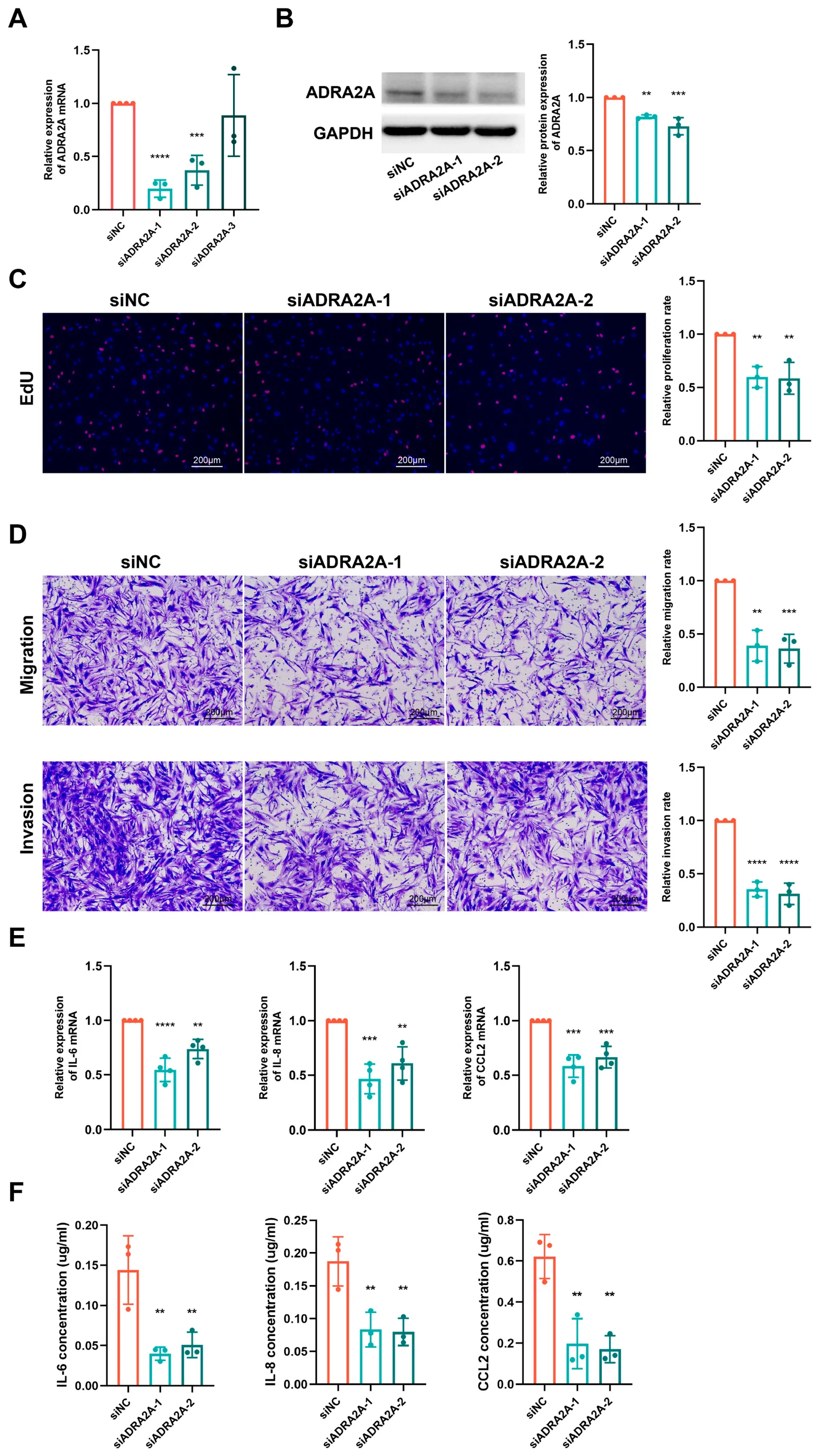
Effect of ADRA2A knockdown on the biological functions of RA FLS. (**A**,**B**) The ADRA2A-siRNA knockdown efficiency verification by RT-qPCR and Western blot. Two siRNAs (siADRA2A-1 and siADRA2A-2) with stable knockdown efficiency were selected for subsequent functional experiments. (**C**) Cell proliferation was assessed using EdU incorporation assays. Representative images showing EdU-positive proliferating cells and DAPI-stained nuclei are presented, with the corresponding quantification of proliferation rates shown in the right panel. Original magnification, ×100. (**D**) Transwell assays were performed to evaluate the chemotactic migration and invasion of RA FLSs. Relative migration and invasion rates were calculated by counting the cells that had migrated or invaded in each group, and then normalizing the values to those of the siNC group. Representative images and corresponding quantitative analyses are shown. Original magnification, ×100. (**E**,**F**) RT-qPCR and ELISA were performed to quantify the mRNA and secretion levels of proinflammatory cytokines. Quantitative analyses of relative cytokine expression are shown. Data are presented as the means ± SD of at least 3 independent experiments. ** *p* < 0.01, *** *p* < 0.001, and **** *p* < 0.0001 versus siNC by Student’s *t*-test. ADRA2A, Adrenoceptor Alpha 2A; PHGDH, Phosphoglycerate Dehydrogenase; RA, rheumatoid arthritis; FLS, fibroblast-like synoviocytes; NC, negative control; RT-qPCR, Quantitative real-time PCR; EDU, 5-ethynyl-2′-deoxyuridine; DAPI, 4′,6-Diamidino-2-phenylindole.

To determine whether ADRA2A mediates the regulatory effects of PHGDH on RA FLS functions, we first examined the expression of ADRA2A in RA FLS by RT-qPCR (Figure 3E) and Western blot (Figure 3F) and in synovial tissue by IHC (Figure 3G). The results showed that the expression pattern of ADRA2A was consistent with that of PHGDH, with both significantly upregulated in RA FLS and synovial tissues at both the levels of mRNA and protein. Moreover, scRNA-seq analysis revealed that ADRA2A expression was almost restricted to FLS (Figure 3H). We then further investigated the effect of ADRA2A on the biological functions of RA FLS by knocking it down using siRNA. Firstly, ADRA2A was effectively knocked down by siADRA2A-1 and siADRA2A-2 verified by RT-qPCR (Figure 4A). The subsequent Western blot assay confirmed the decreased protein level of ADRA2A (Figure 4B). The results of cell experiments demonstrated that ADRA2A knockdown phenocopied the effects of PHGDH knockdown, manifested as suppressed proliferation (Figure 4C), migration, invasion (Figure 4D), and inflammatory response (Figure 4E,F) of RA FLS.

**Figure 5:**
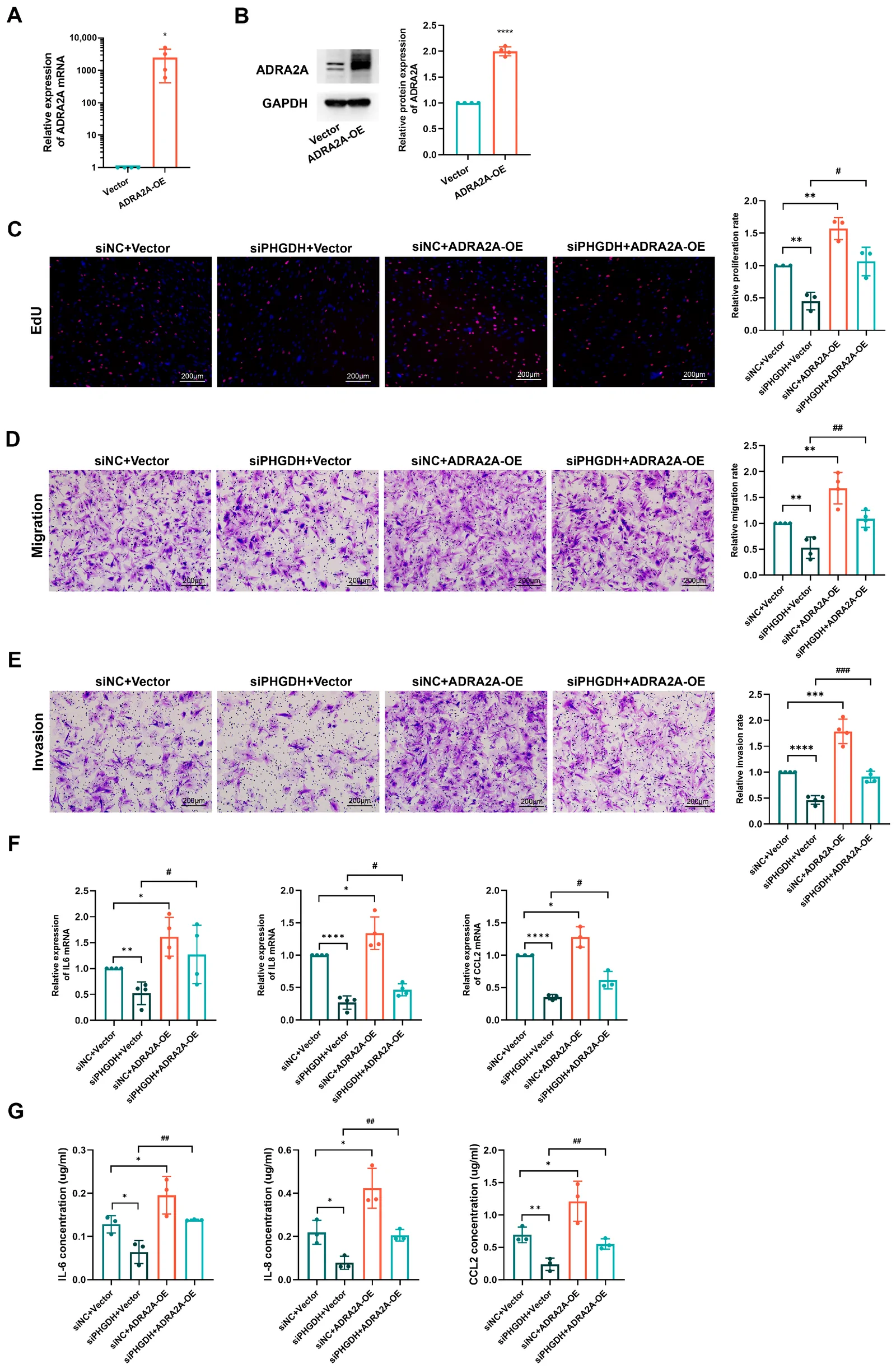
ADRA2A overexpression rescues the inhibitory effects of PHGDH knockdown on RA FLS proliferation, migration, and invasion. (**A**,**B**) ADRA2A overexpression model was established in RA FLS using lentiviral transduction, and the overexpression efficiency was confirmed by RT-qPCR and Western blot. (**C**) ADRA2A overexpression increased the proportion of EdU-positive proliferating RA FLS compared with the vector group. ADRA2A overexpression under PHGDH knockdown partially restored the proportion of proliferating cells compared with the siPHGDH + Vector group. Representative images of EdU-positive proliferating cells and DAPI-stained nuclei are shown, with corresponding quantitative analysis. Original magnification, ×100. (**D**,**E**) ADRA2A overexpression increased RA FLS migration and invasion compared with the vector group. ADRA2A overexpression under PHGDH knockdown partially restored the migratory and invasive ability of RA FLS compared with the siPHGDH + Vector group. Original magnification, ×100. (**F**,**G**) RT-qPCR and ELISA was performed to quantify the mRNA and secretion levels of proinflammatory cytokines. Data are presented as the mean ± SD from at least three independent experiments. * *p* < 0.05, ** *p* < 0.01, *** *p* < 0.001, and **** *p* < 0.0001 versus Vector or siNC + Vector; ^#^ *p* < 0.05, ^##^ *p* < 0.01, and ^###^ *p* < 0.001 versus siPHGDH + Vector, as determined by Student’s *t*-test. ADRA2A, Adrenoceptor Alpha 2A; PHGDH, Phosphoglycerate Dehydrogenase; RA, rheumatoid arthritis; FLS, fibroblast-like synoviocytes; NC, negative control; RT-qPCR, Quantitative real-time PCR; EDU, 5-ethynyl-2′-deoxyuridine; DAPI, 4′,6-Diamidino-2-phenylindole.

To further illustrate that PHGDH regulates the biological functions of FLSs through ADRA2A, we performed rescue experiments using ADRA2A over-expression lentivirus with prior PHGDH knockdown. We first verified the over-expression efficiency of ADRA2A lentivirus by qRT-PCR and Western blot. ADRA2A was significantly over-expressed at both mRNA (Figure 5A) and protein levels (Figure 5B). Notably, rescue experiments showed that overexpression of ADRA2A significantly reversed the inhibitory effects of PHGDH knockdown on the proliferation (Figure 5C), migration (Figure 5D), invasion (Figure 5E) and proinflammation (Figure 5F,G) of RA FLS. Collectively, these findings indicate that PHGDH exerts its regulatory role on the biological functions of RA FLS by modulating the expression of ADRA2A.

### 3.5. Targeting PHGDH Reduces Severity of Arthritis in Rats with Collagen-Induced Arthritis (CIA)

**Figure 6:**
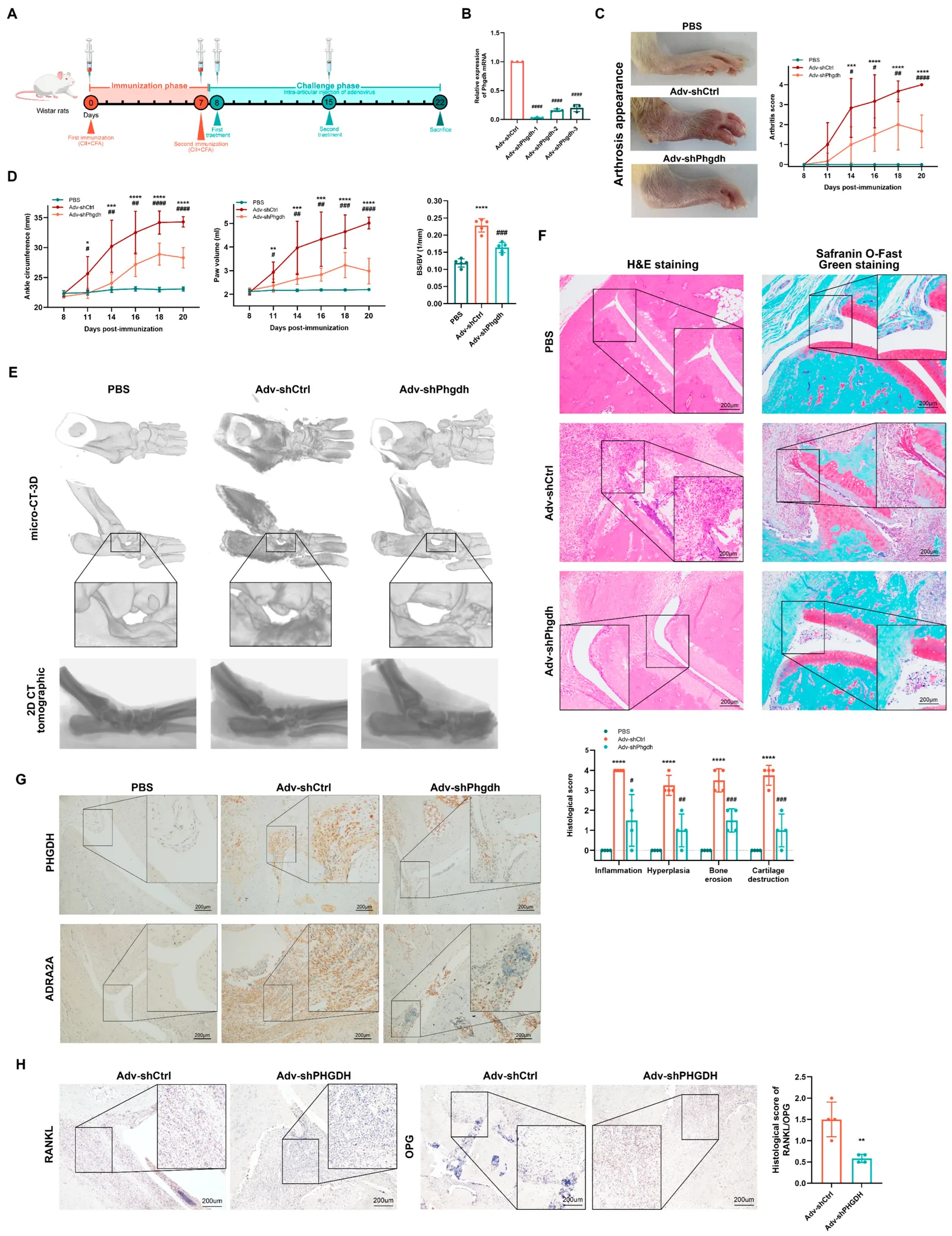
PHGDH knockdown alleviates arthritis severity and joint destruction in CIA rats. (**A**) Schematic illustration of the collagen-induced arthritis (CIA) model and adenovirus treatment schedule. Wistar rats were immunized with CII/CFA on days 0 and 7, followed by intra-articular injection of adenovirus during the challenge phase on days 8 and 15. (**B**) The knockdown efficiency of Adv-shPhgdh was confirmed by RT-qPCR, and Adv-shPhgdh-1 was selected for subsequent in vivo experiments. (**C**) Representative images of hind paws and arthritis scores in PBS-, Adv-shCtrl-, and Adv-shPhgdh-treated CIA rats. PHGDH knockdown reduced arthritis severity compared with the Adv-shCtrl group. (**D**) PHGDH knockdown decreased ankle circumference and paw volume compared with the Adv-shCtrl group. (**E**) Representative micro-CT 3D reconstruction and 2D CT images of ankle joints. Quantitative analysis showed that PHGDH knockdown attenuated bone destruction in CIA rats. (**F**) Representative H&E staining and Safranin O–Fast Green staining of ankle joint sections. PHGDH knockdown reduced synovial inflammation, synovial hyperplasia, bone erosion, and cartilage destruction compared with the Adv-shCtrl group. Original magnification, ×100. (**G**) IHC staining showed that PHGDH and ADRA2A expression was increased in synovial tissues of Adv-shCtrl-treated CIA rats, whereas PHGDH knockdown reduced the expression of PHGDH and ADRA2A. (**H**) IHC staining showed that RANKL expression was increased and OPG was decreased Adv-shCtrl-treated than Adv-shPHGDH-treated CIA rats. The corresponding RANKL/OPG histological score was quantified. Original magnification, ×100. Data are presented as the mean ± SD. * *p* < 0.05, ** *p* < 0.01, *** *p* < 0.001, and **** *p* < 0.0001 versus PBS; ^#^ *p* < 0.05, ^##^ *p* < 0.01, ^###^ *p* < 0.001, and ^####^ *p* < 0.0001 versus Adv-shCtrl, as determined by Student’s *t*-test. ADRA2A, Adrenoceptor Alpha 2A; PHGDH, Phosphoglycerate Dehydrogenase; CIA, collagen-induced arthritis; RT-qPCR, Quantitative real-time PCR; IHC, immunohistochemistry.

Finally, we further validated the role of PHGDH in vivo. A rat model of CIA was successfully established with twice immunization of CII + CFA individually on day 0 and day 7, and Phgdh-shRNA lentivirus was administered via intra-articular injection on day 8 and day 15 for intervention (Figure 6A). We first confirmed the knockdown efficiency of Rat-Phgdh adenovirus (Adv-shPhgdh) (Figure 6B). Compared with the control group (Adv-Ctrl), the Adv-shPhgdh group exhibited significantly reduced joint swelling and arthritis scores (Figure 6C,D). Micro-CT analysis also revealed improved bone structural parameters, including bone volume fraction (BS/BV) (Figure 6E). Histopathological evaluation of joint tissues by H&E staining and Safranin O/Fast Green staining further demonstrated that intra-articular knockdown of PHGDH effectively alleviated synovial inflammation, synovial hyperplasia, and cartilage and bone destruction (Figure 6F). To assess the impact of PHGDH on osteoclastogenesis, we examined RANKL and OPG expression in the synovium. PHGDH knockdown significantly suppressed RANKL while concurrently elevating OPG expression (Figure 6G), suggesting a protective effect against osteoclastogenesis. Furthermore, immunohistochemical (IHC) analysis revealed that ADRA2A expression was downregulated in the synovial tissues of the Adv-shPhgdh group (Figure 6H). The results showed that ADRA2A expression was downregulated in the synovial tissues of the Phgdh-shRNA group (Figure 6G). These findings are consistent with the in vitro results, further supporting that PHGDH exerts its pathogenic role in RA through the regulation of ADRA2A.

## 4. Discussion

This study is the first to report that PHGDH is highly expressed in both RA FLS and RA synovial tissues, with predominant localization in the synovial lining and sublining layers. Functional experiments demonstrated that PHGDH knockdown significantly inhibited the migration, invasion, and proliferation abilities of RA FLS and reduced the expression levels of inflammatory factors. These results suggest that PHGDH plays a critical regulatory role in the aberrant activation of RA FLS and may represent a potential new target for RA intervention.

PHGDH, the first rate-limiting enzyme in the de novo serine synthesis pathway, has garnered extensive attention in the oncology field in recent years. Studies have shown that PHGDH is upregulated in various malignant tumors, supporting tumor cell proliferation and survival by promoting serine synthesis [6,7,12], and its inhibitors have demonstrated promising antitumor activity in PHGDH-overexpressing tumor models [13,14,15]. Beyond its metabolic function, PHGDH is also involved in non-enzymatic transcriptional regulation. For instance, in late-onset Alzheimer’s disease (LOAD), PHGDH can inhibit autophagy and accelerate amyloid pathology by regulating the transcription of IKKα and HMGB1 [7]. In this study, through RNA-seq, RT-qPCR, and Western blot validation, we found that PHGDH positively regulates the mRNA and protein expression of ADRA2A in RA FLS, suggesting that PHGDH may influence RA FLS function through a non-metabolic pathway.

ADRA2A is a member of the adrenergic receptor family, known to be involved in regulating neurotransmitter release and sympathetic nervous system activity [16,17]. At the signal transduction level, ADRA2A reduces cAMP levels by inhibiting adenylate cyclase, thereby regulating multiple downstream signaling pathways, including PKA, PKC, and MAPK [18,19,20,21,22]. In recent years, the function of ADRA2A in diseases has shown significant context dependency: In pancreatic ductal adenocarcinoma (PDAC), downregulation of ADRA2A expression is significantly correlated with lymph node metastasis, high pathological grade, advanced disease, and poor prognosis [23], suggesting it may play a tumor-suppressive role. In contrast, in colorectal cancer, adrenergic stimulation promotes tumor growth via ADRA2A/Gi-mediated YAP activation [16]. Furthermore, α2-AR agonists have demonstrated potent antitumor activity in various immunocompetent tumor models, an effect dependent on ADRA2A expression in host cells [24]. These findings indicate that the function of ADRA2A is specific to tissue and disease type.

Based on the results of this study, we hypothesize that in the context of RA, PHGDH may participate in the regulation of FLS activation by upregulating ADRA2A expression. As a metabolic enzyme, PHGDH’s function is no longer confined to metabolic regulation; it may also play a role in the inflammation and tissue destruction of autoimmune diseases by influencing key molecules such as ADRA2A. Currently, intervention strategies targeting RA FLS have made preliminary progress in clinical translation. For example, the cyclin-dependent kinase inhibitor seliciclib has entered Phase Ib clinical trials (TRAFIC study), and the monoclonal antibody ASP5094 targeting integrin α9 on FLS has completed Phase IIa clinical trials. Although the primary efficacy endpoint was not met, these efforts have provided practical experience for translational exploration targeting FLS surface molecules [25,26]. In recent years, strategies using cytotoxic aptamers for the specific elimination of RA FLS have also shown therapeutic potential in preclinical models [27]. Therefore, further mechanistic research and interventional exploration centered on the PHGDH-ADRA2A axis hold significant scientific value and clinical translational potential.

This study has certain limitations. First, although we observed the regulatory effect of PHGDH on ADRA2A, the specific underlying mechanism requires further elucidation. Second, whether PHGDH participates in RA FLS function through its enzymatic activity or a non-enzymatic mechanism needs to be validated in depth using enzyme activity mutants and conditional gene knockout animal models. Third, while our loss-of-function data support a role for PHGDH in RA pathogenesis, complementary gain-of-function experiments in FLS with low basal PHGDH expression are needed to determine whether PHGDH upregulation alone is sufficient to drive pathogenic phenotypes such as proliferation, migration, invasion, and inflammatory cytokine production. Future research will focus on the functional mechanism of the PHGDH-ADRA2A axis in the development and progression of RA, and evaluate its feasibility as a novel therapeutic target for RA.

## 5. Conclusions

We found that increased PHGDH promotes the inflammatory responses, proliferation and aggression of RA FLS through upregulating ADRA2A expression. Targeting PHGDH may provide a novel therapeutic approach for RA.

## Data Availability

The datasets generated during and/or analyzed during the current study are available from the corresponding author on reasonable request.

## Supplementary Materials

The following supporting information can be downloaded at: https://www.mdpi.com/article/10.3390/cells15161501/s1, Figure S1: Representative Western blots and densitometric analysis of PHGDH and ADRA2A expression in RA FLS transfected with siNC or siPHGDH and treated with PBS or L-serine (0.4mM) for 72h. ADRA2A expression was normalized to α-tubulin. Data are presented as the mean ± SD from three independent experiments. Statistical significance was assessed using two-tailed Student’s *t*-test. **** *p* < 0.0001; Table S1: Demographic and clinical characteristics of patients with active RA; Table S2: Sequences of primers for RT-qPCR; Table S3: Sequences of siRNAs and shRNAs; Sequence S1: Sequences of h-ADRA2A overexpression lentivirus.
