# Supplementary material for: PHGDH Promotes Synovial Aggression and Inflammation via Upregulating ADRA2A Expression in Rheumatoid Arthritis
Source: Cells. 2026 Aug 20;15(16):1501. doi: 10.3390/cells15161501 (PMC13511596; doi:10.3390/cells15161501)
Supplement: Supplementary file 1 [file cells-15-01501-s001.zip › cells-4453092-supplementary.pdf]

**Table S1**. Demographic and clinical characteristics of patients with active RA.

| Demographic and clinical characteristics | Values |
|---|---|
| Age, yrs (mean ± SD) | 57.7 ± 10.4 |
| Female, n (%) | 17 (85.0) |
| Male, n (%) | 3 (15.0) |
| Disease duration, yrs (mean ± SD) | 7.8 ± 12.2 |
| Rheumatoid factor-positive, n (%) | 17 (85.0) |
| Anti-CCP-positive, n (%) | 16 (80) |
| DAS28-ESR (mean ± SD) | 5.5 ± 1.4 |
| Previous medications, n (%) | |
| Prednisone (<10mg/d) | 14 (70.0) |
| Methotrexate | 18 (90.0) |
| Leflunomide | 3 (15.0) |
| Hydroxychloroquine | 15 (75.0) |

**Table S2**. Sequences of primers for RT-qPCR.

| NCBI Gene Symbol | Forward Primer | Reverse Primer |
|---|---|---|
| h-ACTB | CATGTACGTTGCTATCCAGGC | CTCCTTAATGTCACGCACGAT |
| h-PHGDH | CTGCGGAAAGTGCTCATCAGT | TGGCAGAGCGAACAATAAGGC |
| h-ADRA2A | TCGTCATCATCGCCGTGTTC | AAGCCTTGCCGAAGTACCAG |
| h-IL1B | ATGATGGCTTATTACAGTGGCAA | GTCGGAGATTCGTAGCTGGA |
| h-IL6 | ACTCACCTCTTCAGAACGAATTG | CCATCTTTGGAAGGTTCAGGTTG |
| h-IL8 | ACTGAGAGTGATTGAGAGTGGAC | AACCCTCTGCACCCAGTTTTC |
| h-CCL2 | CAGCCAGATGCAATCAATGCC | TGGAATCCTGAACCCACTTCT |
| Rat-Actb | CGATATCGCTGCGCTCGT | ATACCCACCATCACACCCTGG |
| Rat-Phgdh | TCCTCGTCATGAACACCCCC | TTCCCAGTGTCTTCCCGTTC |

**Table S3**. Sequences of siRNAs and shRNAs.

| siRNA | | Sequence (5'-3') |
|---|---|---|
| h-siPHGDH-1 | sense | CGCAGAACUCACUUGUGGAAU(dT)(dT) |
| | anti-sense | AUUCCACAAGUGAGUUCUGCG(dT)(dT) |
| h-siPHGDH-2 | sense | CAGGCUUGCUGAAUGACAA(dT)(dT) |
| | anti-sense | UUGUCAUUCAGCAAGCCUG(dT)(dT) |
| h-siPHGDH-3 | sense | CAGACUUCACUGGUGUCAGAU(dT)(dT) |
| | anti-sense | AUCUGACACCAGUGAAGUCUG(dT)(dT) |
| h-siADRA2A-1 | sense | CGCACGCUCUUCAAAUUCU(dT)(dT) |
| | anti-sense | AGAAUUUGAAGAGCGUGCG(dT)(dT) |
| h-siADRA2A-2 | sense | GCAUAAUUGCCUUUUCCUA(dT)(dT) |
| | anti-sense | UAGGAAAAGGCAAUUAUGC(dT)(dT) |
| h-siADRA2A-3 | sense | CGCUGUAAAUAUACACUAU(dT)(dT) |
| | anti-sense | AACUCCAACCGCUUCUUCC(dT)(dT) |
| Rat-shPhgdh-1 | Target Sequence | GGACAGAGCTGAACGGAAGA |
| Rat-shPhgdh-2 | Target Sequence | GCACCTTTGCCCAGTGCAAGA |
| Rat-shPhgdh-2 | Target Sequence | GCCCACTATGATCGGCCTACT |

**Supplementary Sequence S1**. Sequences of h-ADRA2A overexpression lentivirus.

GGCCGTTTTTGGCTTTTTTGTTAGACGAAGCTTGGGCTGCAGGTCGACTCTAGAGGATCCCGCCAC
CATGTTCCGCCAGGAGCAGCCGTTGGCCGAGGGCAGCTTTGCGCCCATGGGCTCCCTGCAGCCG
GACGCGGGCAACGCGAGCTGGAACGGGACCGAGGCGCCGGGGGGCGGCGCCCGGGCCACCCC
TTACTCCCTGCAGGTGACGCTGACGCTGGTGTGCCTGGCCGGCCTGCTCATGCTGCTCACCGTGT
TCGGCAACGTGCTCGTCATCATCGCCGTGTTCACGAGCCGCGCGCTCAAGGCGCCCCAAAACCTC
TTCCTGGTGTCTCTGGCCTCGGCCGACATCCTGGTGGCCACGCTCGTCATCCCTTTCTCGCTGGCC
AACGAGGTCATGGGCTACTGGTACTTCGGCAAGGCTTGGTGCGAGATCTACCTGGCGCTCGACGT
GCTCTTCTGCACGTCGTCCATCGTGCACCTGTGCGCCATCAGCCTGGACCGCTACTGGTCCATCAC
ACAGGCCATCGAGTACAACCTGAAGCGCACGCCGCGCCGCATCAAGGCCATCATCATCACCGTGT
GGGTCATCTCGGCCGTCATCTCCTTCCCGCCGCTCATCTCCATCGAGAAGAAGGGCGGCGGCGGC
GGCCCGCAGCCGGCCGAGCCGCGCTGCGAGATCAACGACCAGAAGTGGTACGTCATCTCGTCGT
GCATCGGCTCCTTCTTCGCTCCCTGCCTCATCATGATCCTGGTCTACGTGCGCATCTACCAGATCGC
CAAGCGTCGCACCCGCGTGCCACCCAGCCGCCGGGGTCCGGACGCCGTCGCCGCGCCGCCGGG
GGGCACCGAGCGCAGGCCCAACGGTCTGGGCCCCGAGCGCAGCGCGGGCCCGGGGGGCGCAG
AGGCCGAACCGCTGCCCACCCAGCTCAACGGCGCCCCTGGCGAGCCCGCGCCGGCCGGGCCGC
GCGACACCGACGCGCTGGACCTGGAGGAGAGCTCGTCTTCCGACCACGCCGAGCGGCCTCCAGG
GCCCCGCAGACCCGAGCGCGGTCCCCGGGGCAAAGGCAAGGCCCGAGCGAGCCAGGTGAAGCC
GGGCGACAGCCTGCCGCGGCGCGGGCCGGGGGCGACGGGGATCGGGACGCCGGCTGCAGGGC
CGGGGGAGGAGCGCGTCGGGGCTGCCAAGGCGTCGCGCTGGCGCGGGCGGCAGAACCGCGAG
AAGCGCTTCACGTTCGTGCTGGCCGTGGTCATCGGAGTGTTCGTGGTGTGCTGGTTCCCCTTCTTC

TTCACCTACACGCTCACGGCCGTCGGGTGCTCCGTGCCACGCACGCTCTTCAAATTCTTCTTCTGG
TTCGGCTACTGCAACAGCTCGTTGAACCCGGTCATCTACACCATCTTCAACCACGATTTCCGCCGCG
CCTTCAAGAAGATCCTCTGTCGGGGGGACAGGAAGCGGATCGTGGGTATGGACTACAAGGATGA

**Supplementary Figure S1**

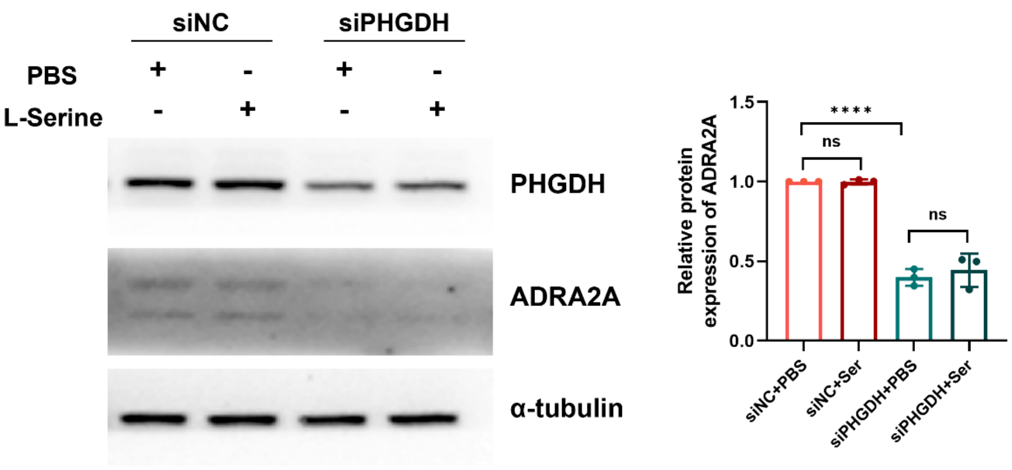


**Supplementary Figure S1.** Representative Western blots and densitometric analysis of PHGDH and ADRA2A expression in RA FLS transfected with siNC or siPHGDH and treated with PBS or L-serine (0.4mM) for 72h. ADRA2A expression was normalized to α-tubulin. Data are presented as the mean ± SD from three independent experiments. Statistical significance was assessed using two-tailed Student's t-test. ****$P$ < 0.0001.
